# Deoxycholic Acid Triggers NLRP3 Inflammasome Activation and Aggravates DSS-Induced Colitis in Mice

**DOI:** 10.3389/fimmu.2016.00536

**Published:** 2016-11-28

**Authors:** Shengnan Zhao, Zizhen Gong, Jiefei Zhou, Chunyan Tian, Yanhong Gao, Congfeng Xu, Yingwei Chen, Wei Cai, Jin Wu

**Affiliations:** ^1^Department of Pediatric Surgery, Xinhua Hospital, Shanghai Jiaotong University School of Medicine, Shanghai, China; ^2^Shanghai Institute for Pediatric Research, Shanghai Jiaotong University School of Medicine, Shanghai, China; ^3^Shanghai Key Laboratory of Pediatric Gastroenterology and Nutrition, Shanghai, China; ^4^State Key Laboratory of Proteomics, National Center for Proteomics Science, Beijing Institute of Radiation Medicine, Beijing, China; ^5^National Engineering Research Center for Protein Drugs, Beijing, China; ^6^Department of Geriatrics, Xinhua Hospital, Shanghai Jiaotong University School of Medicine, Shanghai, China; ^7^Shanghai Institute of Immunology, Institutes of Medical Sciences, Shanghai Jiaotong University School of Medicine, Shanghai, China

**Keywords:** high-fat diet, bile acid, inflammation, inflammasome, IL-1β, inflammatory bowel disease

## Abstract

A westernized high-fat diet (HFD) is associated with the development of inflammatory bowel disease (IBD). High-level fecal deoxycholic acid (DCA) caused by HFD contributes to the colonic inflammatory injury of IBD; however, the mechanism concerning the initiation of inflammatory response by DCA remains unclear. In this study, we sought to investigate the role and mechanism of DCA in the induction of inflammation *via* promoting NLRP3 inflammasome activation. Here, we, for the first time, showed that DCA dose-dependently induced NLRP3 inflammasome activation and highly pro-inflammatory cytokine-IL-1β production in macrophages. Mechanistically, DCA-triggered NLRP3 inflammasome activation by promoting cathepsin B release at least partially through sphingosine-1-phosphate receptor 2. Colorectal instillation of DCA significantly increased mature IL-1β level in colonic tissue and exacerbated DSS-induced colitis, while *in vivo* blockage of NLRP3 inflammasome or macrophage depletion dramatically reduced the mature IL-1β production and ameliorated the aggravated inflammatory injury imposed by DCA. Thus, our findings show that high-level fecal DCA may serve as an endogenous danger signal to activate NLRP3 inflammasome and contribute to HFD-related colonic inflammation. NLRP3 inflammasome may represent a new potential therapeutical target for treatment of IBD.

## Introduction

A westernized high-fat diet (HFD) is associated with the development of diverse inflammatory diseases, including inflammatory bowel disease (IBD). Epidemiological studies indicate that HFD consumption, as an important environmental factor, could increase the risk of both ulcerative colitis and Crohn’s disease (1, 2). Increasing evidence shows that prolonged exposure to the high level of fecal bile acids, which is caused by HFD, contributes to the occurrence of IBD and gastrointestinal cancer (3–5). Deoxycholic acid (DCA) makes up 58% of bile acid in human feces, and dietary fat is observed to mainly increase fecal secondary bile acids, especially DCA, which further increases the concentration of colonic DCA (6, 7). Stenman and colleagues found that a diet high in fat increased the fecal concentration of DCA nearly 10-fold (7). Furthermore, high level of DCA, which is comparable to its concentration in feces of high-fat-fed mice could disrupt epithelial integrity and is related to barrier dysfunction (8, 9). Meanwhile, transient colorectal instillation of DCA in rat leads to mild colonic inflammation, whereas long-term feeding of mice with a diet supplemented with DCA, which mimic the effect of a HFD, induces obvious colonic inflammation and injury that resembles human IBD (10, 11). These findings support the potential role of excessive fetal DCA in mediating colonic inflammatory injury of IBD; however, the mechanism concerning the initiation of inflammatory response by DCA remains largely unclear.

The innate immune system provides the first line to recognize microbes or endogenous molecules *via* pathogen-associated molecular patterns (PAMPs) or damage-associated molecular patterns (DAMPs) by host pattern recognition receptors (PRRs). Inflammasome is a major component of innate immunity, and recent studies have highlighted the critical role of NLRP3 inflammasome in the inflammatory response. NLRP3 inflammasome is a molecular platform that can be activated by multiple PAMPs or DAMPs and thus involved in diverse inflammatory diseases (12–14). Upon activation, NLRP3 recruits apoptosis-associated speck-like protein (ASC) and caspase-1 (interleukin-1 converting enzyme, ICE), leading to the maturation and secretion of highly pro-inflammatory cytokines, such as IL-1β (15). Unlike other cytokines, bioactive IL-1β production relies on inflammasome activation (16–18). More importantly, emerging evidences suggest the pivotal role of NLRP3 inflammasome in the development and pathogenesis of IBD (19). Single nucleotide polymorphisms of nlrp3 gene have been linked to the development of Crohn’s disease (20). NLRP3 as well as caspase-1-deficient mice were protected from DSS-induced colitis (21, 22). Consistently, clinical studies show increased IL-1β level in the serum and inflamed colonic tissues of IBD patients, and IL-1β levels are correlated well with the severity of intestinal inflammation and disease activity (23–26). Furthermore, pharmacological inhibition of IL-1β or Caspase-1 was shown to successfully ameliorate intestinal inflammation in colitis animal models (27, 28).

Given the important role of the inflammasome in intestinal immunity, we hypothesized that NLRP3 inflammasome activation may be involved in the DCA-induced colonic inflammation. In this study, we provide evidence that DCA can activate NLRP3 inflammasome and induce obvious mature IL-1β production in macrophages by promoting cathepsin B release at least partially *via* S1PR2 receptors. Colorectal instillation of DCA in mice strongly aggravates DSS-induced colitis and caspase-1 inhibition as well as macrophage depletion substantially alleviates colonic inflammation and injury.

## Materials and Methods

### Reagents

Lipopolysaccharide (LPS), DCA, CA-074 Me, *N*-acetyl-l-cysteine (NAC), and JTE-013 were purchased from Sigma-Aldrich (St. Louis, MO, USA). Poly (dA:dT) was obtained from Invivogen (San Diego, CA, USA). Nigericin was purchased from Cayman Chemical (Ann Arbor, MI, USA). *Z*-Guggulsterone was obtained from Santa cruz Biotechnology (Santa Cruz, CA, USA). VX-765 (belnacasan) was purchased from Selleck (Houston, TX, USA). 2′,7′-dichlorofluorescein diacetate (DCF-DA) was from Invitrogen/molecular probes. RPMI 1640, DMEM, and antibiotics were obtained from Invitrogen (Carlsbad, CA, USA). ELISA Kits were purchased from eBioscience (San Diego, CA, USA).

### Mice

The 6- to 8-week-old C57BL/6 female mice were purchased from Experimental Animal Center of the Chinese Academy of Sciences (Shanghai, China) and housed in a specific pathogen-free (SPF) facility. The animal study protocols complied with the Guide for the Care and Use of Medical Laboratory Animals issued by the Ministry of Health of China and approved by the Shanghai Laboratory Animal Care and Use Committee.

### Cells

The murine macrophage cell line J774A.1 was obtained from Type Culture Collection of the Institutes of Biomedical Sciences, Fudan University (Shanghai, China). J774A.1 cells were cultivated in DMEM culture medium (Invitrogen) supplemented with 10% fetal bovine serum (Gibico) and 1% penicillin/streptomycin (Invitrogen) at 37°C with 5% CO_2._ Bone marrow-derived macrophages (BMDMs) were isolated and cultured as described elsewhere (29). Briefly, bone marrow cells were harvested from femurs and tibiae of C57BL/6 mice. Cells were then cultured in DMEM supplemented with 10% FBS and 30% L929 cell-conditioned medium (as a source of M-CSF) for 6–7 days. Adherent cells were used in the following experiments.

### *In Vitro* DCA Treatment

J774A.1 cells or BMDMs were primed with 1 μg/ml LPS for 5 h before stimulation with DCA at different concentrations, then, supernatants (SNs) were harvested at indicated time points and the IL-1β level was determined by ELISA Kit (eBioscience) according to the manufacturer’s instructions. For some experiments, various inhibitors (e.g., NAC, CA-074 Me) were added to the culture medium 30 min ahead of DCA treatment.

### *Salmonella* Infection

J774A.1 cells (1 × 10^6^) were infected for 1 h with the *Salmonella* (1:100) and then cultured in fresh medium supplemented with gentamicin (100 μg/ml).

### Lysosome and Cathepsin B Imaging

Lipopolysaccharide-primed J774A.1 cells were incubated with or without DCA (100 μM, 24 h); then, the cells were stained with Lyso Tracker Green DND-26 (Invitrogen) or cathepsin B fluorogenic substrate z-Arg-Arg cresyl violet (Neuromics) for 1 h, followed by Hoechst staining for half an hour. Fluorogenic signals were captured by inverted fluorescence microscope (Leica).

### Reactive Oxygen Species Measurement

Lipopolysaccharide-primed J774A.1 cells were treated with or without DCA (100 μM), and nigericin stimulation (20 μM) was regarded as positive control. ROS production was measured by using DCF-DA (Invitrogen) probes according to the manufacturer’s instructions. Briefly, cells were incubated with DCF-DA (15 μM) for 1 h at 37°C after DCA stimulation. Fluorescence was visualized directly under a fluorescence microscope.

### ICP-OES Assay

Lipopolysaccharide-primed J774A.1 cells (1 × 10^7^) were treated with or without DCA (100 μM, 24 h); then, the cells were lysed in ultra pure nitric acid before microwave digestion and then diluted to 5% HNO_3_. Intracellular K^+^ was analyzed by using Perkin Elmer Optima 8000 ICP-OES Spectrometer. External K calibration was performed between 0 and 10 ppm.

### Transfection of Small Interfering RNA Oligonucleotides

J774A.1 cells in 6-well plates were transfected with NLRP3, TGR5 small interfering RNA, or scrambled siRNA by using TransIT-Jurkat (Mirus Bio, Madison, WI, USA), followed by LPS stimulation and DCA treatment (100 μM, 24 h). IL-1β in supernatant was measured by ELISA. RNA oligonucleotides sequences were as follows: NLRP3, forward 5′-GGC GAG ACC UCU GGG AAA ATT-3′ and reverse 5′-UUU UCC CAG AGG UCU CGC CTT-3′; TGR5, forward 5′-CUG GAA CUC UGU UAU CGC UTT-3′ and reverse 5′-AGC GAU AAC AGA GUU CCA GTT-3′.

### Western Blot

J774A.1 cells were lysed by protein lysis buffer (Sigma) containing protease and phosphatase inhibitors (Theromo), and the cell culture supernatant was concentrated by acetone precipitation. Cell lysates (50 μg) or concentrated supernatant proteins were resolved by SDS-PAGE, transferred to PVDF membranes (0.2 μm), and probed with antibodies against IL-1β (Cell Signaling Technologies), Caspase-1 (Santa Cruz, CA, USA), NLRP3 (R&D), TGR5 (Abcam), and β-actin (sigma). For the detection of cytosolic cathepsin B, cells were thoroughly washed and permeabilized with extraction buffer containing 50 μg/ml digitonin for 15 min at 4°C to lyse the plasma membrane without disturbing the intracellular membranes. These cell lysates were then subjected to SDS-PAGE and immunoblotted for cathepsin B (Santa Cruz, CA, USA). Reactive signals were detected by ECL Western Blotting Substrate (Thermo Fisher Scientific, Waltham, MA, USA) and ChemiDoc™ XRS^+^ System (Bio-Rad).

### Cytosolic Cathepsin B Activity Assay

Extraction of cytosolic protein was performed as described above and cathepsin B activity was determined by a fluorometric assay kit (ApexBio). Briefly, 50 μl of each sample (containing 100 μg of total protein) was incubated with cathepsin B reaction buffer (50 μl) and substrate Ac-RR-AFC (10mM, 2 μl) at 37°C for 1 h, and free amino-4-trifluoromethyl coumarin (AFC) was measured through a fluorescence spectrophotometer with an excitation wavelength of 400 nm and an emission wavelength of 505 nm.

### Colitis Induction and Treatment

Acute colitis was induced in C57BL/6 mice with 2.5% DSS (MP Biomedicals) dissolved in drinking water given *ad libitum* for 7 days. DSS-treated animals were randomly divided into three groups and received an enema of PBS, 4mM DCA (in PBS, 0.1 ml), or 4mM DCA plus intraperitoneal injection of caspase-1 inhibitor (belnacasan, 50 mg/kg/day), respectively, for seven consecutive days from day 1 of DSS treatment (*n* = 7 in each group). Body weight was measured daily throughout the course of experiment. On day 8, mice were sacrificed and colon length was measured. The paraffin sections of colon tissues were stained with hematoxylin and eosin. A scoring system was applied to assess diarrhea and the presence of occult or overt blood in the stool. Colon homogenates were used for immunoblot analysis of mature IL-1β and assessment of MPO activity.

### *In Vivo* Macrophages Depletion

To evaluate the role of macrophages in the colonic inflammation exacerbated by DCA, colitis was induced in C57BL/6 mice with 2.5% DSS for 7 days as described above. Macrophages depletion was performed by intraperitoneal injection of 0.2 ml clodronate-liposomes (www.clodronateliposomes.com, Netherlands) 4 days prior to DSS treatment and on days 0, 2, 4, and 6 during DSS treatment as described elsewhere (30). Animals were randomly divided into five groups, including control group, DSS-treated group, DSS-macrophages depletion group, DSS-treated plus DCA enema group and DSS-treated plus DCA enema-macrophages depletion group (*n* = 7 in each group). On day 8, mice were sacrificed for sample collection and analysis as mentioned above.

### Histological Analysis

Colonic histological scoring was determined by inflammatory cell infiltration (0–3) and tissue damage (0–3) in a blinded manner. For tissue inflammation, increased numbers of inflammatory cells in the lamina propria were scored as 1, confluence of inflammatory cells extending into the submucosa as 2, and transmural extension of the infiltrate as 3. For tissue damage, discrete lymphoepithelial lesions were scored as 1, mucosal erosions were scored as 2, and extensive mucosal damage and/or extension into deeper structures of the bowel wall were scored as 3. The combined histological score ranged from 0 to 6.

### Statistics

All results were expressed as mean ± SEM. Statistical significance was assessed by two-tailed Student’s *t*-test or one-way analysis of variance (ANOVA). Differences were considered statistically significant at *p* < 0.05.

## Results

### DCA Induces Caspase-1 Activation and IL-1β Maturation in Macrophages

**Figure 1:**
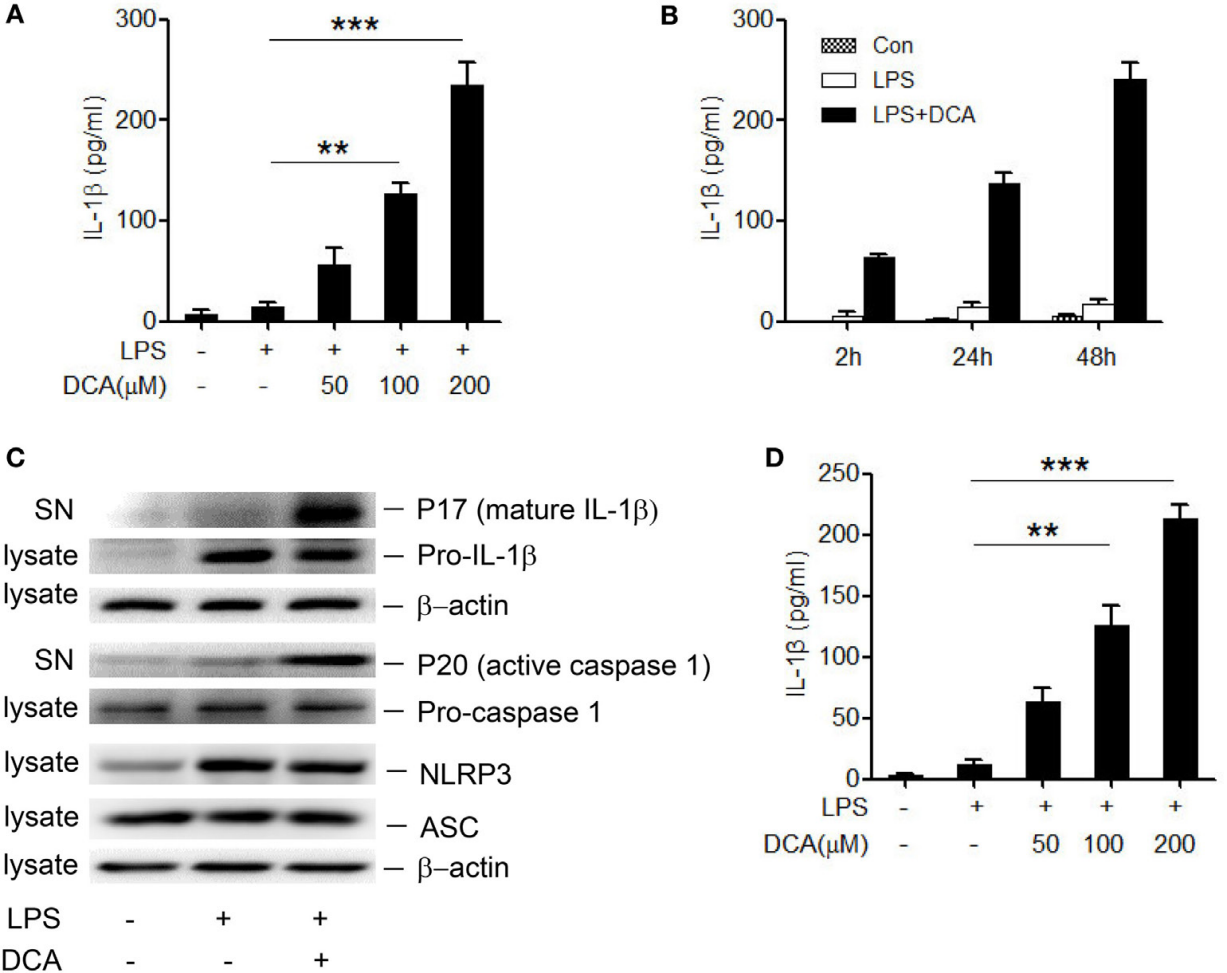
**DCA induces mature IL-1β release from macrophages**. **(A)** LPS-primed J774A.1 macrophages were treated with DCA (0, 50, 100, and 200 μM) for 24 h. Released IL-1β in culture medium was analyzed by ELISA. **(B)** LPS-primed J774A.1 macrophages were treated with DCA (100 μM) for different time courses. Released IL-1β was analyzed by ELISA. **(C)** Immunoblot analysis of mature IL-1β (mIL-1β, 17 kD), active caspase-1 (p20, 20 kD) in culture supernatants (SN) and precursors of IL-1β (pro-IL-1β), caspase-1 (pro-caspase-1), NLRP3, ASC in cell lysates of LPS-primed J774A.1 macrophages treated with DCA (100 μM) for 24 h. β-actin was regarded as a loading control. **(D)** ELISA analysis of IL-1β in supernatants from LPS-primed bone marrow-derived macrophages (BMDMs) treated with different dosage of DCA. **p < 0.01; ***p < 0.001. Error bars indicate SEM. Representative data from at least three independent experiments giving similar results are shown.

The NLRP3 inflammasome recognizes many endogenous materials as danger signals and triggers the release of strong pro-inflammatory cytokines including active IL-1β, thus contributing to diverse diseases as atherosclerosis, Alzheimer’s disease, and T2 diabetes (31, 32). Here, we hypothesized that DCA may also exert its inflammatory potential *via* activating inflammasomes. Therefore, LPS-primed murine macrophage cell line J774A.1 was treated with different dosage of DCA, and the result showed that IL-1β secretion was obviously induced in a dose- and time-dependent manner in response to DCA (Figures 1A,B). IL-1β maturation was further confirmed by the detection of cleaved IL-1β and active caspase-1 as assessed by western blotting (Figure 1C). Meanwhile, DCA also dose-dependently induced IL-1β maturation and secretion in murine bone marrow-derived macrophages (BMDMs) (Figure 1D). Moreover, we treated macrophages with DCA after LPS challenge and observed that DCA stimulation did not significantly increase the pro-IL-1β, NLRP3, and ASC level (Figure 1C), and DCA alone had no obvious effect on pro-IL-1β and NLRP3 expression either (data not shown). These data demonstrate that DCA could induce IL-1β maturation and release in macrophages through promoting inflammasome activation.

### DCA Activates the NLRP3 Inflammasome

**Figure 2:**
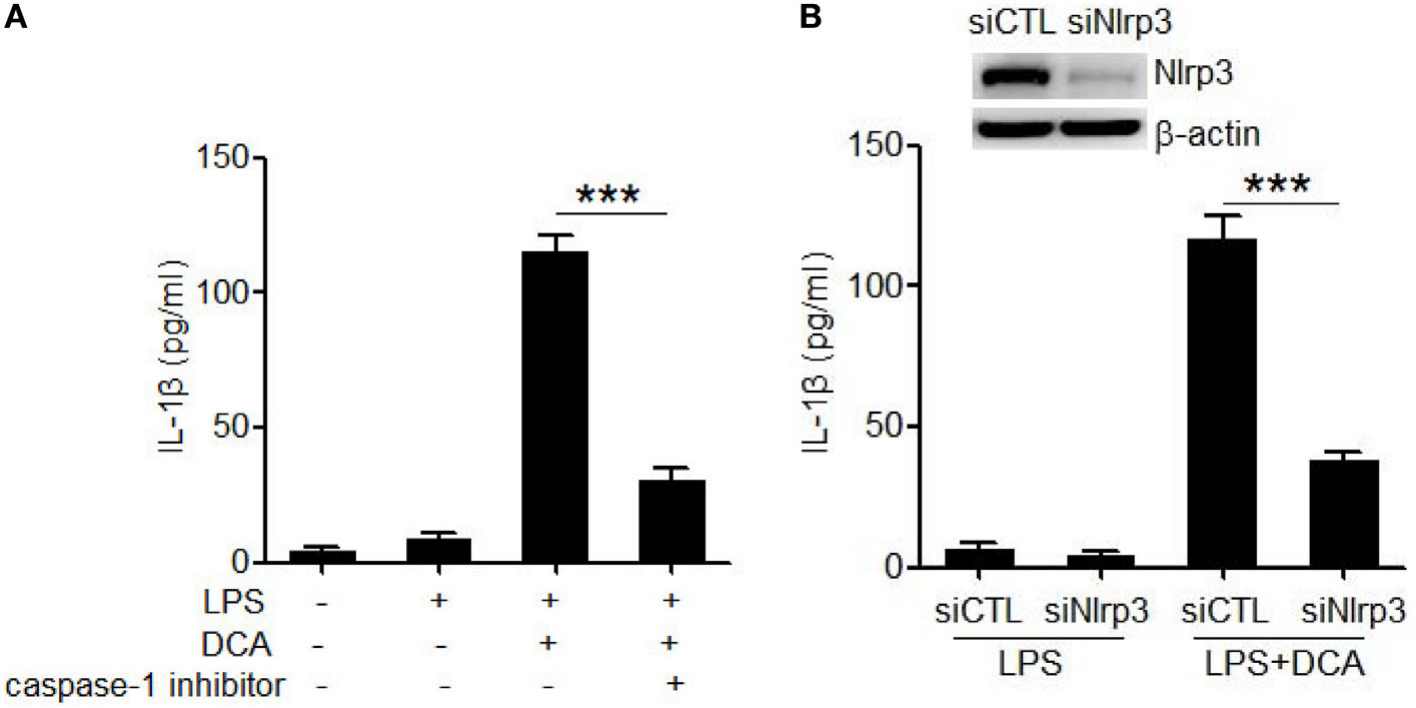
**DCA activates the NLRP3 inflammasome**. **(A)** LPS-primed J774A.1 macrophages were treated with DCA (100 μM) in the presence or absence of caspase-1 inhibitor belnacasan (10 μM). IL-1β in supernatants was analyzed by ELISA. **(B)** Control siRNA (siCTL) or Nlrp3 siRNA (siNlrp3)-transfected J774A.1 macrophages were primed with LPS and then treated with DCA (100 μM) for 24 h. IL-1β in supernatants was analyzed by ELISA. Inset, immunoblot analysis of NLRP3 expression in siCTL or siNlrp3-transfected J774A.1 cells. ***p < 0.001. Error bars indicate SEM. The data shown are from three independent experiments.

Caspase-1 activation is an essential step to produce and release of mature IL-1β during inflammasome activation (13, 14). Here, caspase-1 inhibitor (belnacasan) significantly impaired the IL-1β secretion induced by DCA (Figure 2A), indicating that DCA induces IL-1β maturation in a caspase-1-dependent manner. In order to investigate whether DCA can activate the NLRP3 inflammasome, which is usually involved in caspase-1 activation induced by multiple danger signals, we knocked down the expression of NLRP3 by transfecting macrophages with siRNA specific for Nlrp3. We found that LPS-primed J774A.1 macrophages transfected with control siRNA produced large amounts of IL-1β upon DCA exposure. In contrast, macrophages transfected with Nlrp3 siRNA produced much less IL-1β in response to DCA or nigericin (Figure 2B; Figure S1 in Supplementary Material), indicating the requirement of NLRP3 for IL-1β processing. Meanwhile, Nlrp3 siRNA transfection had no effect on IL-1β production induced by *Salmonella* or poly(dA:dT) (Figure S1 in Supplementary Material). These results suggest that DCA at least partially activates the NLRP3 inflammasome, leading to the activation of caspase-1 and subsequent IL-1β maturation and release.

### DCA-Induced NLRP3 Inflammasome Activation Requires Cathepsin B Release

**Figure 3:**
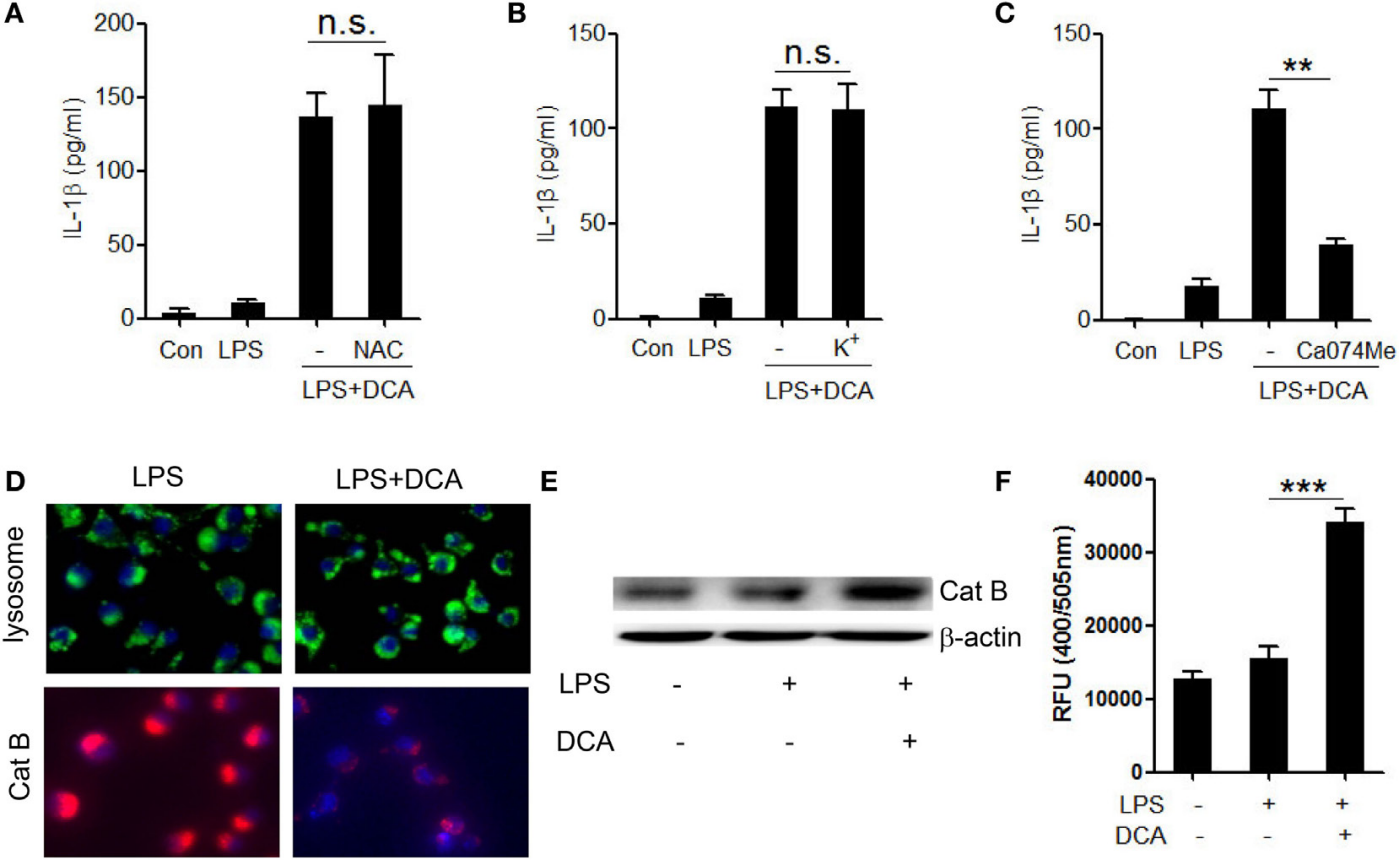
**Activation of NLRP3 inflammasome by DCA depends on cathepsin B release**. **(A)** LPS-primed J774A.1 macrophages were treated with DCA in the presence or absence of NAC (10 mM) for 24 h. IL-1β in supernatants was analyzed by ELISA. **(B)** LPS-primed J774A.1 macrophages were treated with DCA in the presence or absence of KCL (140 mM) for 2 h. IL-1β in supernatants was analyzed by ELISA. **(C)** LPS-primed J774A.1 macrophages were treated with DCA in the presence or absence of CA-074Me (50 μM) for 24 h. IL-1β in supernatants was measured by ELISA. **(D)** LPS-primed J774A.1 macrophages were treated with or without DCA. The cells were incubated with Lyso Tracker (green) or cathepsin B (Cat B) substrate z-Arg-Arg-cresyl violet, which emits red signal upon cleavage by cathepsin B, followed by Hoechst staining (blue). **(E,F)** LPS-primed J774A.1 macrophages were treated with or without DCA, then, cytosolic protein was isolated for the immunoblot analysis of mature cathepsin B **(E)** as well as fluorometric assay of cathepsin B activity **(F)**. **p < 0.01; ***p < 0.001. n.s., no statistically significant difference (*p* > 0.05). Error bars indicate SEM. The data shown are representative of three individual experiments.

Three major cellular events are regarded as common mechanisms for the NLRP3 inflammasome activation, including potassium (K^+^) efflux, reactive oxygen species (ROS) formation, and cathepsin B leakage (33–36). To identify the upstream events involved in DCA-induced inflammasome activation, we observed the effect of selective inhibitors on IL-1β secretion in DCA-treated macrophages. DCA-induced IL-1β secretion was not suppressed by the inhibition of ROS formation and K^+^ efflux (Figures 3A,B), which was confirmed by the fact that DCA stimulation had no obvious effect on intracellular ROS formation and potassium level (Figure S2 in Supplementary Material); however, it was dramatically blunted by CA-074Me, a cathepsin B inhibitor (Figure 3C). Consistently, DCA treatment strongly decreased the staining of lysosomal cathepsin B (Figure 3D) and increased cytosolic cathepsin B level as well as its activity (Figures 3E,F), which indicated the release of cathepsin B into cytoplasm. Unexpectedly, lysosomes retained their morphology in DCA-treated cells (Figure 3D), implying the involvement of other mechanisms responsible for the DCA-induced cathepsin B release. These data suggest that DCA induces NLRP3 inflammasome activation mainly through promoting cathepsin B release.

### DCA-Induced Cathepsin B Release and IL-1β Production Are Mediated by Sphingosine-1-Phosphate Receptors 2

**Figure 4:**
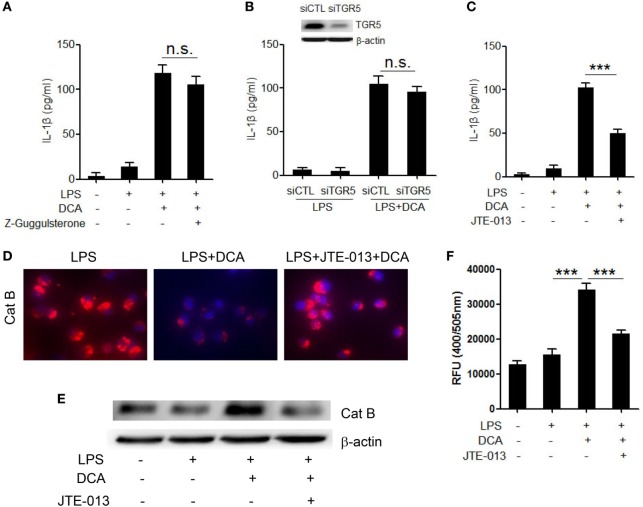
**DCA-induced cathepsin B release and IL-1β secretion is mediated by S1PR 2**. **(A)** LPS-primed J774A.1 macrophages were treated with DCA in the presence or absence of *Z*-Guggulsterone (20 μM). IL-1β in supernatants was analyzed by ELISA. **(B)** Control siRNA (siCTL) or TGR5 siRNA (siTGR5)-transfected J774A.1 macrophages were primed with LPS and then treated with DCA. IL-1β in supernatants was analyzed by ELISA. Inset, immunoblot analysis of TGR5 expression in siCTL or siTGR5-transfected J774A.1 cells. **(C)** LPS-primed J774A.1 macrophages were treated with DCA in the presence or absence of JTE-013 (10 μM). IL-1β in supernatants was analyzed by ELISA. **(D)** LPS-primed J774A.1 macrophages were treated with DCA in the presence or absence of JTE-013. The cells were incubated with cathepsin B substrate followed by Hoechst staining. **(E,F)** LPS-primed J774A.1 macrophages were treated with DCA in the presence or absence of JTE-013, and cytosolic protein was isolated for the immunoblot analysis of mature cathepsin B **(E)** as well as fluorometric assay of cathepsin B activity **(F)**. ***p < 0.001; n.s., no statistically significant difference (*p* > 0.05). Error bars indicate SEM. The data shown are representative of three individual experiments giving similar results.

To better understand the molecular mechanism underlying the induction of NLRP3 inflammasome activation by DCA, the major bile acid nuclear receptor farnesoid X-receptor (FXR) and membrane receptor TGR5 were selectively inhibited (37). Unexpectedly, neither suppression of FXR nor knockdown of TGR5 expression had obvious effect on mature IL-1β production in response to DCA (Figures 4A,B; Figure S3 in Supplementary Material), which indicated the involvement of other bile acid receptors. Sphingosine-1-phosphate receptor 2 (S1PR 2), which belongs to G protein-coupled receptor (GPCR), is reported to be another kind of bile acid receptor that appears to play an important role in the regulation of hepatic lipid metabolism (38). Importantly, S1PR2 is found as the major S1PRs expressed in monocytes/macrophages and also has been required for mast cell degranulation and chemotaxis toward the site of inflammation (39), implying its important role in the immune regulation and inflammatory response. Here, we observed that S1PR2 antagonist JTE-013 could significantly inhibit IL-1β release upon DCA stimulation (Figure 4C); intriguingly, JTE-013 could dramatically prevent cathepsin B release from lysosome (Figures 4D–F), which is critical in the DCA-triggered NLRP3 inflammasome activation. These data strongly suggest that S1PR2 is a key mediator of NLRP3 inflammasome activation induced by DCA.

### DCA Administration Exacerbates DSS-Induced Colitis and Caspase-1 Inhibition Exhibits Significant Protective Role

**Figure 5:**
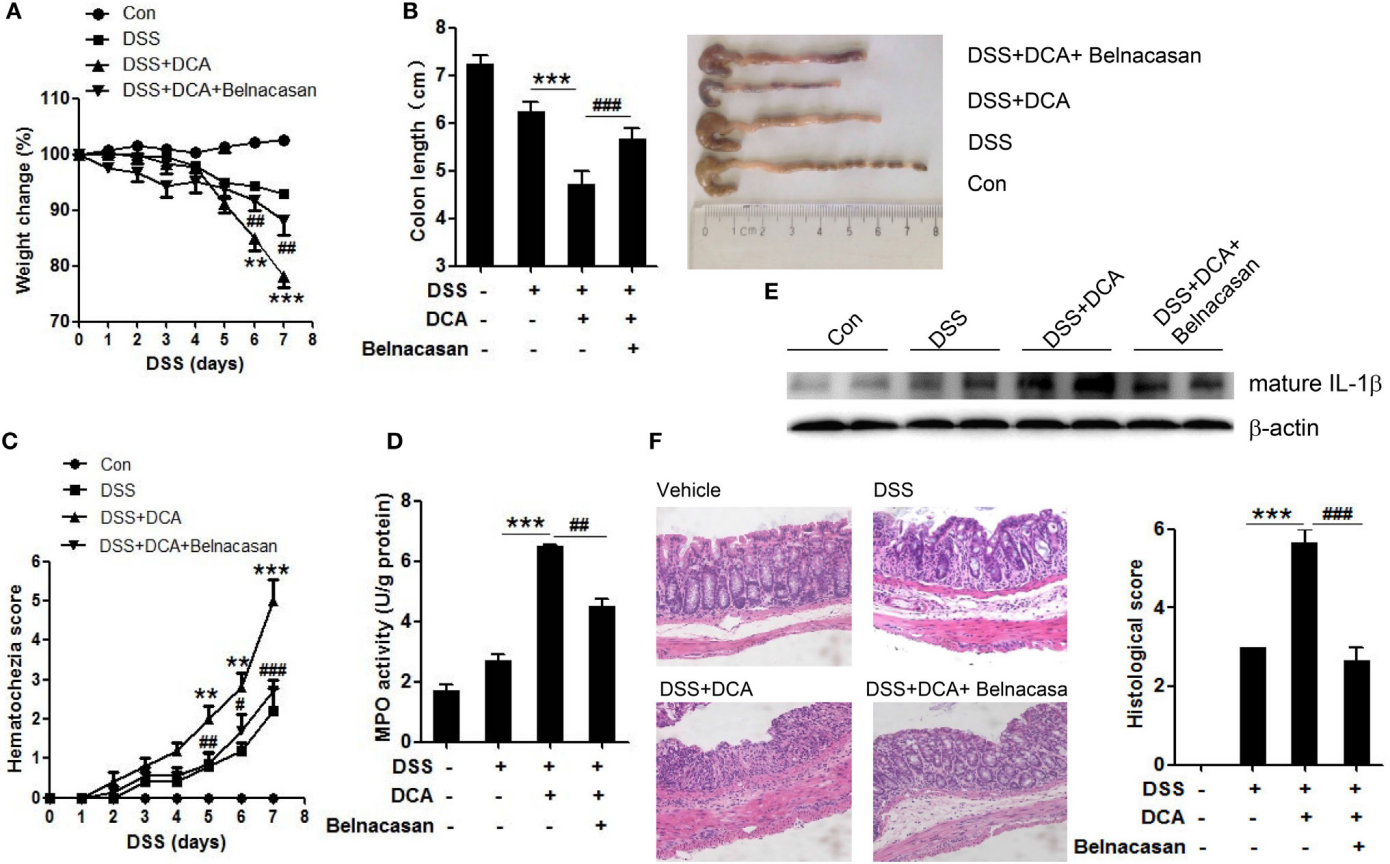
**DCA administration aggravates DSS-induced colitis and caspase-1 antagonist protects mice from severe colon inflammation**. **(A)** Loss of basal body weight, **(B)** colon length, **(C)** haematochezia score, and **(D)** MPO activity in colon tissue of control, DSS-treated, DSS-treated plus DCA enema, DSS-treated plus DCA enema, and belnacasan-injection mice (caspase-1 antagonist, 50 mg/kg/day). **(E)** Immunoblot analysis of mature IL-1β (17 kD) in colonic homogenates, **(F)** H&E staining and histological score of distal colon sections of differently treated mice as described above. **p < 0.01; ***p < 0.001 compared to the DSS-treated alone mice. ^#^*p* < 0.05; ^##^*p* < 0.01; ^###^*p* < 0.001 compared to the DSS-treated plus DCA enema mice. Error bars indicate SEM. The data shown are from three independent experiments.

To investigate the effect of DCA and its inductive role of inflammasome activation in the development of colitis, DSS-treated mice received an enema of 0, 4mM DCA, or 4mM DCA plus intraperitoneal injection of caspase-1 inhibitor belnacasan. The addition of 4mM DCA enema caused much more severe colitis than DSS treatment alone, as evidenced by significant decrease of body weight and shortening of colon length (Figures 5A,B), much higher haematochezia score, and MPO activity (Figures 5C,D). Of note, the mature IL-1β level in colon tissue is elevated dramatically in DCA enema group (Figure 5E), indicating the highly activation of inflammasome. Consistently, H&E staining of colonic tissue in DCA enema group showed significantly higher mucosal inflammatory cell infiltration and more severe epithelial layer destruction compared to that of the DSS alone group (Figure 5F). Indeed, inhibition of caspase-1 with belnacasan obviously reduced the mature IL-1β level in colon tissue and largely prevented the deteriorating role of DCA in the DSS-induced colitis (Figures 5A–F). These data provide further evidence that induction of inflammasome activation is a major pathogenic mechanism of DCA in the colonic inflammation.

### Macrophages Depletion Abrogates the Exacerbating Role of DCA in the DSS-Induced Colitis

**Figure 6:**
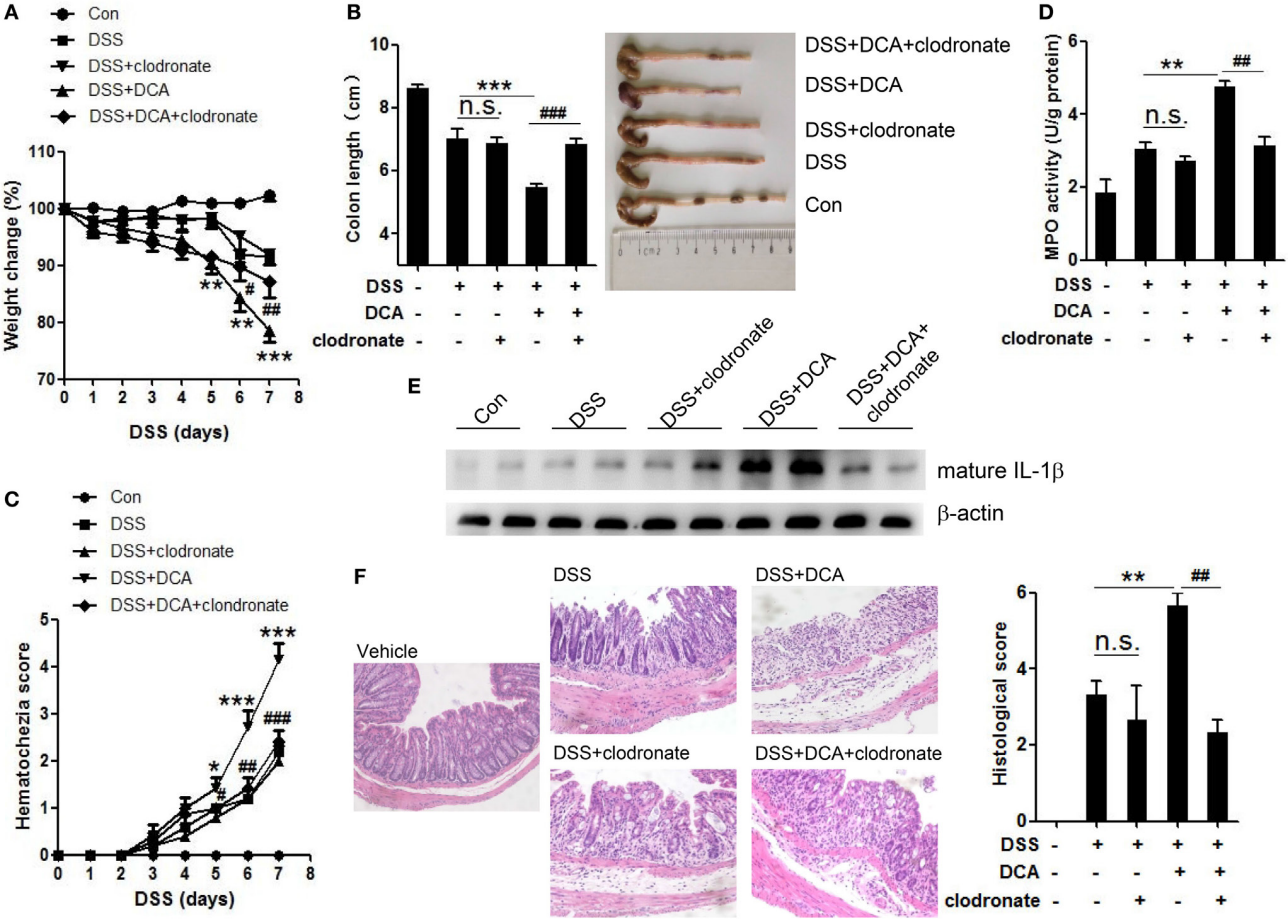
**Depletion of colonic macrophages dramatically reduced the severe inflammation and tissue injury superimposed on DSS-treated mice by DCA**. Clodronate-containing liposomes (CL) was used to eliminate colonic macrophages. **(A)** Loss of basal body weight, **(B)** colon length, **(C)** haematochezia score, and **(D)** MPO activity in colon tissue of control, DSS-treated, DSS-CL, DSS-treated plus DCA enema, and DSS-treated plus DCA enema-CL mice. **(E)** Immunoblot analysis of mature IL-1β (17 kD) in colonic homogenates **(F)** H&E staining and histological score of distal colon sections of differently treated mice as described above. *p < 0.05; **p < 0.01; ***p < 0.001 compared to the DSS-treated alone mice. ^#^*p* < 0.05; ^##^*p* < 0.01; ^###^*p* < 0.001 compared to the DSS-treated plus DCA enema mice. Error bars indicate SEM. Representative data from three independent experiments are shown.

Since IL-1β is primarily produced by activated macrophages, we sought to study whether colonic macrophages are the major effectors of DCA. Macrophage depletion was achieved by injection of clodronate-containing liposomes (CL), which can selectively cause macrophage apoptosis. Immunohistochemistry staining showed mucosal infiltration with large numbers of macrophages in DSS-treated, DCA enema mice, but not clodronate liposomes-administrated counterparts (Figure S3 in Supplementary Material). Macrophage depletion almost abrogated the production of mature IL-1β in colon tissues induced by DCA enema (Figure 6E) and significantly improved the clinical parameters of DSS-treated, DCA enema mice, showing less body weight decease and colon length shortening (Figures 6A,B), much lower haematochezia score, and MPO activity (Figures 6C,D), as well as less mucosal inflammatory cell infiltration and less disruption of the mucosal epithelium (Figure 6F). These data indicate that DCA exerts its pro-inflammatory effect mainly through colonic macrophages.

## Discussion

High-fat diet affects bile acid metabolism, and dietary fat also changes fecal bile acid profile, characterized by substantial increase of DCA in feces, which may contribute to the pathogenesis of IBD; however, the detailed mechanisms still need to be explored. In this study, we, for the first time, proved that DCA could dose-dependently induce NLRP3 inflammasome activation and highly pro-inflammatory cytokine-IL-1β production in macrophages. Knockdown of NLRP3 expression and caspase-1 inhibition largely abrogated DCA-induced IL-1β secretion. *In vivo* experiments showed that colorectal instillation of DCA at the concentration comparable to HFD significantly exacerbated DSS-induced colitis, as evidenced by substantial decrease of body weight, increased histological colitis severity, and importantly, pronounced elevation of mature IL-1β level in colon tissue. Furthermore, blockage of NLRP3 inflammasome activation or macrophage depletion obviously reduced the mature IL-1β level and protected mice from the aggravated inflammation imposed by DCA. Together, our results provide a new mechanism that high level DCA caused by HFD contributes to colonic inflammation through activating NLRP3 inflammasome.

NLRP3 inflammasome, as the most extensively studied inflammasome, can be activated by diverse stimuli, including bacterial toxins, small molecules, as well as various crystals, many of which are host-derived components, such as uric acid, fatty acid, and ATP; here, we demonstrated that secondary bile acid DCA can also activate NLRP3 inflammasome. Despite the structure and characteristic of different stimuli diverse, potassium (K^+^) efflux, cathepsin B leakage from lysosomes, and ROS production are the three common mechanisms of NLRP3 inflammasome activation (33–36). The data obtained in our study demonstrated that NLRP3 inflammasome activation by DCA requires cathepsin B leakage. Numerous studies have shown that cathepsin B leakage usually occurs in the crystal-induced inflammasome activation, such as silica and uric acid crystals (40, 41), accompanied with lysosomal disruption. However, we found that phagocytic uptake was not required for DCA-induced IL-1β secretion (data not shown), and there was no evidence of lysosome damage either, thus indicating the involvement of other mechanisms responsible for the DCA-induced cathepsin B release. Further evidence showed that, instead of the major bile acid receptors (nuclear receptor FXR and membrane receptor TGR5), another kind of GPCR S1PR2 (also known as a bile acid receptor) participated in the DCA-induced IL-1β secretion. In line with our findings, S1PR2 was observed to modulate pro-inflammatory cytokine production (including IL-1β) in bone marrow cells and affect osteoclastogenesis, thus plays an essential role in inflammatory bone loss diseases (42). In addition, Skoura and colleagues proved that S1PR2 expressed in macrophages of atherosclerotic plaque regulates inflammatory cytokine secretion (IL-1β, IL-18) and promotes atherosclerosis (43). Our finding that S1PR2 antagonist dramatically prevented cathepsin B release induced by DCA confirms the role of S1PR2 in mediating NLRP3 inflammasome activation and IL-1β secretion. Therefore, different from the endocytosed crystals, DCA-induced cathepsin B release may be receptor-mediated, although the intermediate process needs further investigation. Blocking S1PR2 signaling might be a novel therapeutic strategy to the HFD-related inflammatory diseases such as atherosclerosis and IBD.

A very recent report exhibited by Guo and colleagues (44) showed that opposite to our findings, bile acids conveyed an inhibitory effect on NLRP3 inflammasome activation. In this report, they demonstrated that bile acids inhibited NLRP3 inflammasome activation *via* the TGR5-cAMP-PKA axis, whereas our data showed that DCA triggered NLRP3 inflammasome activation through S1PR2-cathepsin B pathway, one possible explanation of the discrepancy is that bile acids may have dual regulatory effect on inflammasome activation by different mechanisms. The distinct modulation effect of bile acids on inflammasome activation may depend on diverse factors, such as bile acid concentration, receptors involved, and the presence or absence of other inflammasome activators. Guo et al. proved that bile acids at their physiological concentrations exhibited suppressive effect on inflammasome activation, while our findings suggested that high-level DCA served as a danger signal and contributed to inflammasome activation, which is paralleled with the situation that excessive DCA induced by HFD exacerbated colitis. In addition, the presence or absence of other inflammasome stimuli (such as nigericin, ATP) could be another crucial factor. Similar to the dual effect of bile acids, epinephrine and norepinephrine inhibited cytokine production (IL-1β, IL-6, and TNF-α) in the presence of pro-inflammatory stimuli, otherwise increased such cytokine production through β2 adrenergic receptor (β2AR) (45). Taken together, although the accurate regulation mechanisms of bile acids on inflammasome activation still need to be explored, the role of bile acids may be a double-edged sword and their *in vivo* concentrations should be tightly controlled.

Our *in vivo* studies showed that DCA instillation at a high concentration, which comparable to the concentration on a HFD (7), significantly exacerbated DSS-induced colitis. These results are consistent with previous report that a DSS-induced chronic ulcerative colitis model showed mild inflammatory manifestations and became more aggravated when HFD was administered (46). In our model, DSS affects the epithelial barrier first, which allowing translocated bacteria endotoxin (e.g., LPS) to stimulate lamina propria macrophages and provide the first signal for the synthesis of pro-IL-1β, and subsequently excessive DCA triggers NLRP3 inflammasome activation and promotes bioactive IL-1β secretion, thus contributing to intestinal inflammatory response. These results offer a new explanation for the aggravated inflammatory injury in individuals consuming HFD, who have already suffered from intestinal barrier dysfunction, for example, high meat intake was reported to correlate with increased likelihood of UC relapse (47). In another aspect, numerous *in vitro* and *ex vivo* studies show that high level DCA is toxic to epithelial cells and able to disturb intestinal permeability; meanwhile, mice fed with a diet supplemented with DCA for 10 weeks to mimic HFD exhibits impaired gut barrier function (9), and these findings indicate that long-term exposure to excessive DCA alone can cause intestinal barrier dysfunction and increase the passage of both LPS and DCA into gut mucosa, which may result in the initiation of inflammation at least partially *via* inflammasome activation. It is of certainty that there should be many other factors involved in HFD-induced colitis, including intestinal flora, which also affect bile acids metabolism by increasing secondary bile acids level (such as DCA). The contribution of the interaction between bile acids and intestinal bacteria to the development of HFD-induced colitis remains to be further investigated.

Collectively, our findings disclose that excessive DCA serves as an endogenous danger signal to activate NLRP3 inflammasome in macrophages and exacerbates colonic inflammation. Additionally, increased fecal DCA has also been implicated in the promotion of colon tumorigenesis. DCA was observed to induce colonic crypt cells proliferation and related to colon cancer growth and progression (48–50). Since chronic uncontrolled inflammation in the intestine is closely related to the development of colitis-associated cancer (CAC), our findings here suggest another possible mechanism that DCA participates in the development of colon cancer. In summary, our study offers a plausible mechanistic basis by which HFD might increase the prevalence of colitis and suggests the change of lifestyle to prevent HFD-related inflammatory diseases like IBD.

## Author Contributions

SZ, ZG, and JZ performed research and analyzed data. CT and YG performed research and discussed results. CX analyzed data and discussed results. YC and WC designed and directed research. JW designed and directed research, analyzed data, and wrote the manuscript.

## Conflict of Interest Statement

The authors declare that the research was conducted in the absence of any commercial or financial relationships that could be construed as a potential conflict of interest.
