# Supplementary material for: Deoxycholic Acid Triggers NLRP3 Inflammasome Activation and Aggravates DSS-Induced Colitis in Mice
Source: Front Immunol. 2016 Nov 28;7:536. doi: 10.3389/fimmu.2016.00536 (PMC5124666; doi:10.3389/fimmu.2016.00536)
Supplement: Supplementary file 1 [file Data_Sheet_1.PDF]

Supplementary Data

Deoxycholic acid triggers NLRP3 inflammasome activation and aggravates DSS-induced colitis

Shengnan Zhao, Zizhen Gong, Jiefei Zhou, Chunyan Tian, Yanhong Gao, Congfeng Xu, Yingwei Chen, Wei Cai, Jin Wu

***Address Correspondence to**: Xinhua Hospital, 1665 Kongjiang Road, Shanghai, 200092, China. Phone: +86-21-25076443; Fax: +86-21-65791316; E-mail: wujin51@yahoo.com (J. W.), caiw1978@163.com (W. C.) or way_01chen@hotmail.com (Y.C.)

Inventory of Supplementary Data

Supplementary Figures and Legends

- Supplementary Figure 1. The effect of Nlrp3 siRNA on mature IL-1β secretion induced by nigericin, Salmonella or poly(dA:dT) , related to Figure 2
- Supplementary Figure 2. The effect of DCA treatment on ROS formation and intracellular potassium level in macrophages, related to Figure 3
- Supplementary Figure 3. TGR5 siRNA decreased cAMP formation induced by oleanolic acid (OA) , related to Figure 4
- Supplementary Figure 4. Clodronate-containing liposomes effectively deplete mucosal macrophages infiltration in DSS-treated, DCA-enema mice, related to Figure 6

Supplementary Figures and Legends

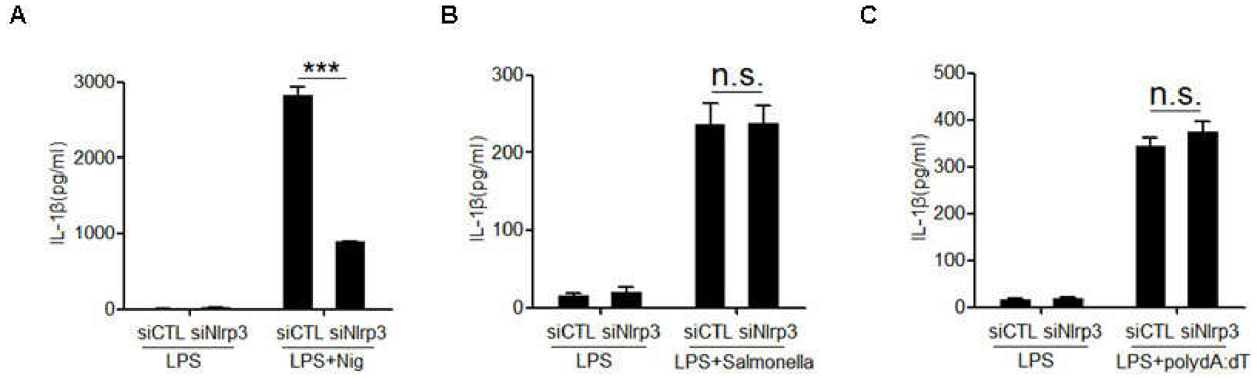

Supplementary Figure 1. The effect of Nlrp3 siRNA on mature IL-1β secretion induced by nigericin, Salmonella or poly(dA:dT). Control siRNA (siCTL) or Nlrp3 siRNA (siNlrp3) transfected J774A.1 macrophages were primed with LPS and then stimulated by (A) nigericin (Nig), (B) Salmonella or (C) poly(dA:dT). IL-1β in supernatants was analyzed by ELISA. ***: $p < 0.001$. n.s.: no statistically significant difference ($p > 0.05$).

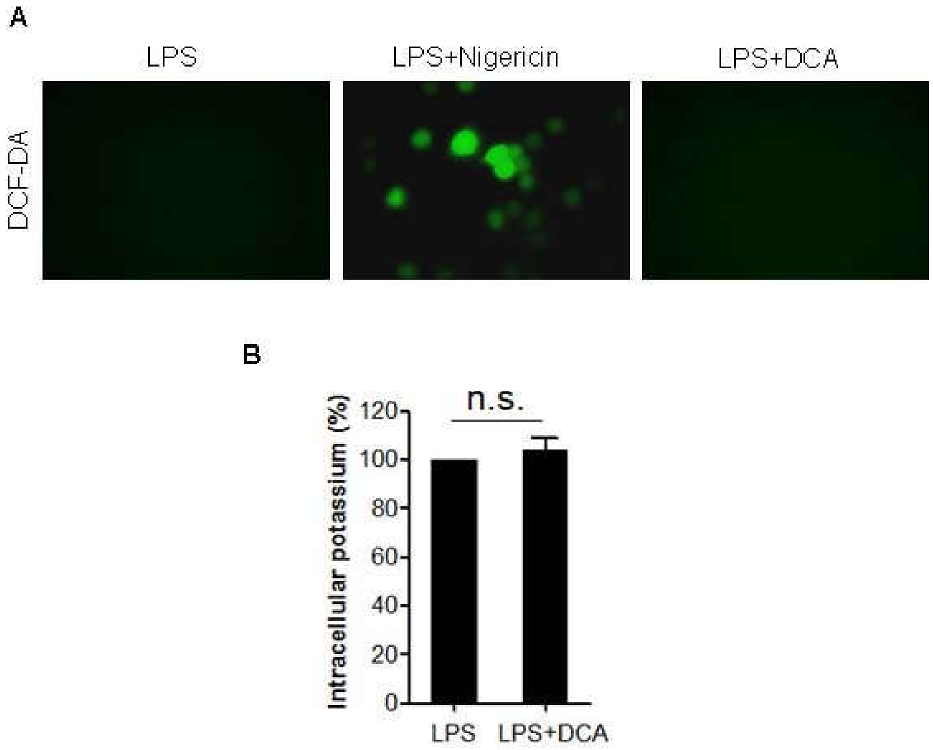

Supplementary Figure 2. The effect of DCA treatment on ROS formation and intracellular potassium level in macrophages. (A) LPS-primed J774A.1 macrophages were treated with nigericin or DCA. Cells were then incubated with DCF-DA probe and fluorescence images was used to exhibit the ROS formation. (B) LPS-primed J774A.1 macrophages were treated with or without DCA, and intracellular potassium level was measured by ICP-OES. n.s.: no statistically significant difference ($p > 0.05$).

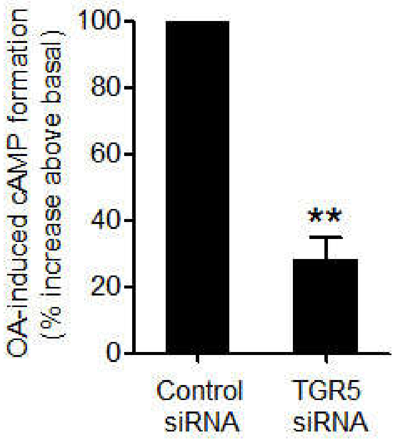

Supplementary Figure 3. TGR5 siRNA decreased cAMP formation induced by oleanolic acid (OA). Control siRNA (siCTL) or TGR5 siRNA (siTGR5) transfected J774A.1 macrophages were treated with OA (10μM) for 1 min and cAMP formation was measured by ELISA. The measurements were expressed as percent increase above basal level. **: $p < 0.01$.

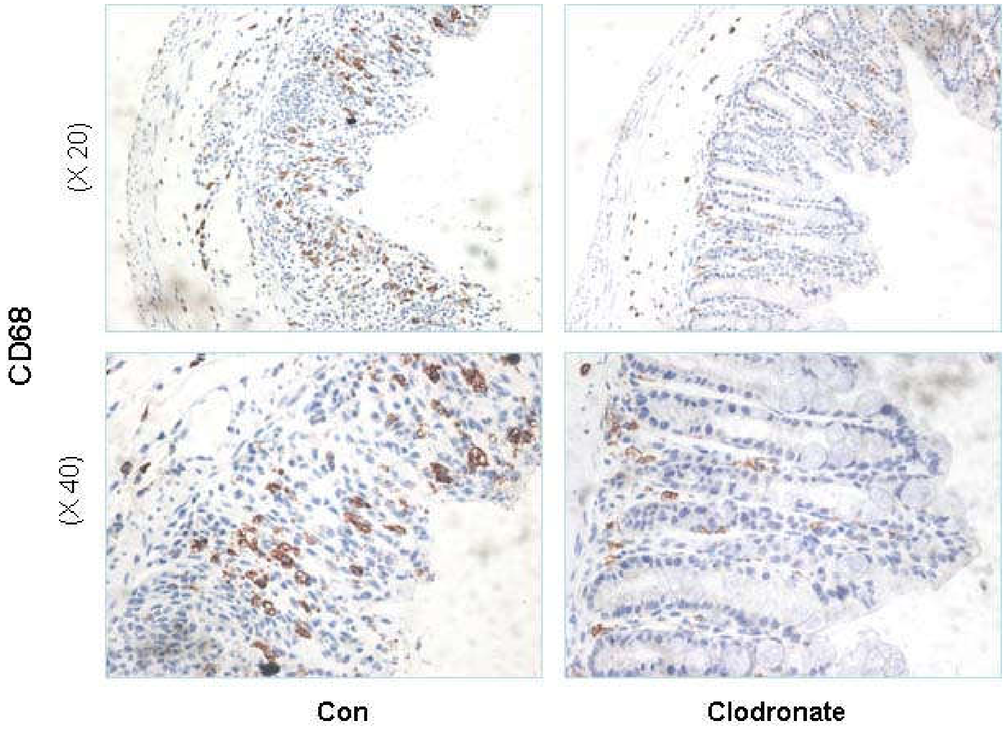

Supplementary Figure 4. Clodronate-containing liposomes effectively deplete mucosal macrophages infiltration in DSS-treated, DCA-enema mice. C57BL/6 mice were given 2.5% DSS for 7 days and 4mM DCA enema was performed simultaneously. PBS (Control) or 0.2ml clodronate-containing liposomes (Clodronate) were intra-peritoneal injected 4 days prior to DSS treatment and on days 0, 2, 4, and 6 during DSS treatment. Mice were sacrificed on day 8 and paraffin sections of colon tissues were stained by immunohistochemistry for macrophages (CD68).
